# Health Care Costs Associated with Ankylosing Spondylitis in Turkey: An Analysis from Nationwide Real-World Data

**DOI:** 10.1155/2013/139608

**Published:** 2013-02-19

**Authors:** Onur Baser, Abdulkadir Burkan, Erdem Baser, Rasim Koselerli, Emre Ertugay, Akif Altinbas

**Affiliations:** ^1^Department of Internal Medicine, Division of Rheumatology, The University of Michigan, Ann Arbor, MI 48109, USA; ^2^STATinMED Research, Istanbul, Turkey; ^3^Social Security Institution, Ankara, Turkey; ^4^Central Bank of Turkey, Ankara, Turkey; ^5^Gastroenterology Clinic, Dıskapı Yıldırım Beyazıt Education and Research Hospital, Ankara, Turkey

## Abstract

*Objectives*. To explore health care costs associated with ankylosing spondylitis (AS) in Turkey. *Methods*. Research-identified data from a system that processes claims for all Turkish health insurance funds were analyzed. Adult prevalent and incident AS patients with two AS visits at least 60 days apart, identified between June 1, 2010 and December 31, 2010, with at least 1 year of continuous health plan enrollment for the baseline and follow-up years were included in the study. Pharmacy, outpatient, and inpatient claims were compiled over the study period for the selected patients. Generalized linear models were used to estimate the expected annual costs, controlling for baseline demographic and clinical characteristics. *Results*. A total of 2.986 patients were identified, of which 603 were incident cases and 2.383 prevalent cases. The mean ages were 39 and 41 years, respectively, and 44% and 38% were women for incident and prevalent cases. Prevalent patients had higher comorbidity scores (5.01 versus 2.24, *P* < 0.001) and were more likely to be prescribed nonsteroidal anti-inflammatory drugs (NSAIDs) (77% versus 72%, *P* < 0.001) or biologics (35% versus 8%, *P* < 0.006) relative to incident patients. Seventy-seven percent of prevalent patients were prescribed NSAIDs, followed by biologic and disease-modifying antirheumatic drugs (DMARDs). Total annual medical costs for incident AS patients were €2.253 and €4.233 for prevalent patients. Pharmacy costs accounted for a significant portion of total costs (88% for prevalent patient, 77% for incident patient), followed by physician office visit costs. Prior comorbidities and treatment type also significantly contributed to overall costs. *Conclusion*. Annual expenditures for AS patients in Turkey were comparable relative to European countries. Pharmaceutical expenditures cover a significant portion of the overall costs. Comparative effectiveness studies are necessary to further decrease health care costs of AS treatment.

## 1. Introduction

Ankylosing spondylitis (AS) is a progressive and chronic inflammatory disease that affects the axial skeleton, causing characteristic back pain that can lead to structural and functional impairments and a decrease in quality of life [1–3].

Epidemiologic studies indicate that AS is a more prevalent disease than previously thought. Overall AS prevalence in Europe is between 0.1% and 1.4% with mid-Europe being 0.3% to 0.5%, and AS incidence is calculated between 0.5 and 14 per 100,000 people per year. [1, 4–6]. There are currently several incidence and prevalence estimates for AS in Turkey. One study among young army recruits estimated AS at 0.14% [7]. Another study estimated the overall prevalence of spondyloarthritis (SpA) including AS at 1.05% in the urban area of Izmir [8]. A study using a survey in western Turkey (Havsa study) found that the prevalence of AS was 0.12% [9]. AS affects two times as many men as women and generally occurs at approximately age 26. The disease is debilitating and can lead to severe functional disability and loss of productivity [10, 11]. 

Prior to the introduction to biologics, due to the low cost of available therapy options, overall treatment costs were relatively low and estimated at *€*2.335 in The Netherlands, *€*2.064 in France, *€*1.572 in Belgium, and *€*1.750 in the United States [2, 11].

Recently, biological DMARDs, including tumor necrosis factor (TNF) inhibitors, have been shown to be efficacious in the treatment of AS. However, this new treatment option is expensive and emphasizes the need for information concerning the current burden of the disease. Although the economic burden of AS has been analyzed in European countries [12, 13], United States [11], Mexico [14], and Canada [15], it is not well documented in Turkey, mainly, due to lack of nationwide data. However, recognizing the importance of information technology and health technology assessment, Turkey has invested in a nationwide integrated system to collect information electronically over the last few years.

This study estimated the medical costs associated with AS in Turkey for both prevalent and incident cases using nationwide real-world claims data.

## 2. Materials and Methods

In 2006, Law 5502 by the Turkish Grand National Assembly aimed to unify the three existing separate social security and health insurance systems (e.g., SSK, Bağ-kur, and Emekli Sandığı) into one, unified Social Security Institute (SSI). Enrollment in the currently existing Universal Health Insurance (UHI) Fund within the SSI will become mandatory, with contribution rates proportional to patients' ability to pay and all beneficiaries entitled to the same benefit package. MEDULA, a claims and utilization management system was established under the 2007 Health Budget Law (SUT), and all public and private facilities under contract with the SSI must submit claims through the MEDULA system. 

MEDULA data was used for this study. MEDULA covers 80% of the population in Turkey and is comprised of pharmacy, inpatient, outpatient, and laboratory claims from 17.800 pharmacies, 5.600 general practitioners, 4.500 medical centers, 1.200 government hospitals, and 338 private hospitals. The remaining 20% of the population not included in the data included people whose contribution rates were paid for by the government due to their income level (this data was maintained separately from the UHI Fund in the SSI until 2012). In addition, members of the Turkish Grand National Assembly and the Supreme Court, as well as foreign insurance policy holders and some military personnel, were excluded from the UHI Fund in the SSI. 

**Figure 1:**
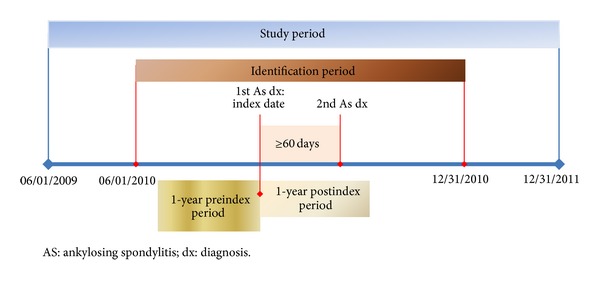
Study period.

All patients diagnosed with AS between age 18 and 99 were included in the study for the identification period (June 1, 2010 through December 31, 2010), using appropriated International Classification of Diseases Tenth Revision Clinical Modification (ICD-10-CM) diagnosis codes. In order to increase reliability of the diagnosis, patients were required to have two AS diagnoses. To ensure that individuals initially evaluated but subsequently not diagnosed with AS were correctly classified as not diagnosed with AS, a minimum of 60 days between claims was required [16]. The date of the first such claim was designated as the index date. All patients were required to have a 1-year preindex (baseline period) and 1-year postindex period (follow-up period) (Figure 1). 

All pharmacy, outpatient, and inpatient claims were compiled for the study period for all remaining patients.

Two groups were analyzed: (1) AS incidence group and (2) AS prevalent group. For the incident group, patients were required to have no AS diagnosis during the baseline period, while patients in the prevalent group were required to have a minimum of one AS diagnosis during the baseline period.

The first aim calculated annual costs of incident and prevalent AS cases in Turkey and identified how total costs were divided by inpatient, outpatient, pharmacy costs, and copayments. Inpatient and outpatient costs were categorized by service type, including surgery, consultations, office visits, laboratory tests, radiology, hospital admissions, medical devices, and other. Patient age and gender were available in the dataset. Since Turkey is divided into seven regions, flags were created according to patients' region of residence. 

To control for clinical characteristics, we calculated a comorbidity index score for each patient for the baseline period, using the Elixhauser method [17]. This index is the sum of a comprehensive set of 30 present comorbid conditions and is widely used in the outcomes research field to determine patient health status [18, 19]. Individual comorbidities such as cardiovascular, diabetes, respiratory disease, and allergies were also examined. 

Generalized linear models (GLMs) were used to estimate risk-adjusted total annual costs for prevalent and incident cases. In particular, the expected annual cost value based on patient demographic and clinical characteristics was estimated. After applying the Park Test, Gamma distribution with log link function was used [20]. According to indications from the Chow test, two separate regression models were run: one for incident cases and the other for prevalent cases [21, 22]. Marginal effects were calculated from estimated coefficients.

All statistical analyses were conducted using SAS V.9.3 and STATA V11.

## 3. Results

A total of 2.986 patients satisfied all inclusion and exclusion criteria, of which 603 were incident cases and 2.383 prevalent cases. As presented in Table 1, most subjects aged 18–39 years (58% of incident cases and 51% of prevalent cases), and 44% of incident cases and 38% of prevalent cases were females. Most subjects resided in the Marmara region (49% for incident cases and 46% for prevalent cases) followed by Central Anatolia and the Aegean region. Prevalent patients had significantly higher comorbid scores relative to incident patients (6.04 versus 2.24, *P* < 0.001). In terms of individual comorbidities, there were no differences in diabetes or allergies, but cardiovascular (16% versus 11%, *P* < 0.001) and respiratory comorbidities (38% versus 24%, *P* < 0.001) were significantly higher for prevalent patients. During the baseline period, 77% (72%) of prevalent (incident) subjects received NSAIDs, 34.75% (7.46%) were prescribed biologics, and 23.29% (21.39%) were prescribed DMARDs. Note that these numbers are not mutually exclusive. 

As Table 2 presents, the total annual cost for prevalent cases was mainly comprised of pharmacy followed by outpatient and inpatient costs and copayments. Total annual costs for incident cases were lower than for prevalent cases. Although lower in terms of value and akin to prevalent cases, total costs for incident cases were mainly comprised of pharmacy costs. However, outpatient and inpatient costs were higher for incident cases relative to prevalent cases.

**Figure 2:**
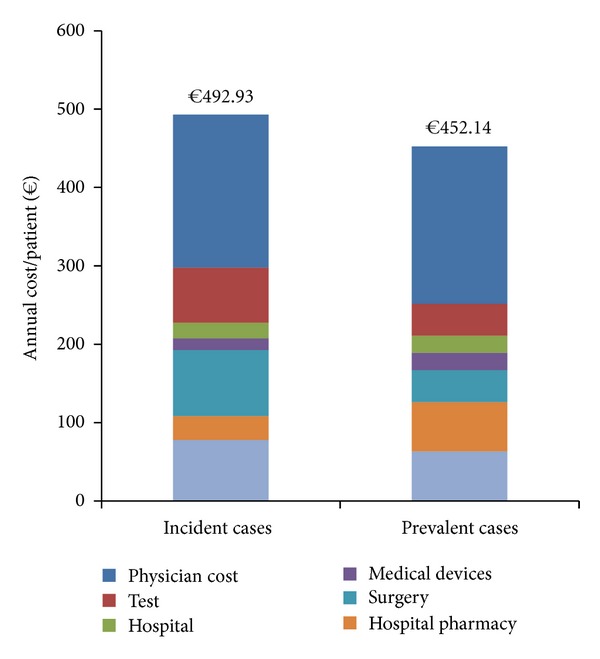
Inpatient and outpatient costs by service.

Figure 2 shows total costs excluding outpatient pharmacy costs and copays. Average annual costs for prevalent and incident cases were *€*452 and *€*492, respectively, after excluding the aforementioned items. For both cases, a significant portion of this cost was due to physician costs (44.31% for prevalent cases and 39.59% for incident cases). For prevalent cases, 14.03% of overall inpatient and outpatient costs were due to other costs, followed by hospital pharmacy (13.88%), laboratory tests (9.06%), surgery (9.01%), medical devices (4.90%), and hospital admissions (4.81%). For incident patients, after physician and surgery costs (17.13%), the most expensive components of inpatient and outpatient costs were other costs (15.86%), followed by laboratory tests (14.29%), inpatient pharmacy (6.11%), hospital admissions (4.02%), and surgery costs (3.00%).

Determinants of total health care costs were estimated using GLMs. After controlling for demographic and clinical factors, risk-adjusted annual total costs were calculated as *€*3.307 for prevalent cases and *€*2.000 for incident cases (Table 3). Prior biologic use significantly contributed to total medical costs for prevalent and incident AS patients (*P* < 0.001). For incidence cases, the cost of care was lower for the 18–39 age group when controlling for other factors. For prevalent cases, there were no differences in health care costs in terms of region, gender, age, comorbidities, or prior NSAID or DMARD use. 

**Figure 3:**
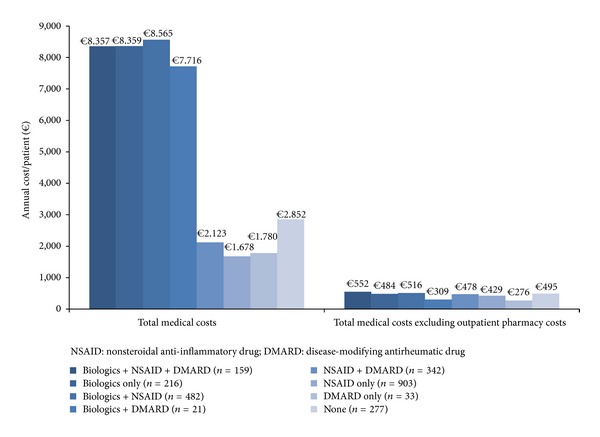
Total annual costs of ankylosing spondylitis prevalent patients by treatment type.

Both models determined that the cost of biologic use was the single most important contributor to overall health care costs.  Figure 3 shows the annual health care costs for prevalent patients according to treatment type. The annual AS cost for prevalent patients prescribed both NSAID and biologics was €8.565. Total health care costs for patients prescribed NSAIDs or DMARDs were lower than for patients who received no treatment (*€*1.678 for NSAID, *€*1.780 for DMARD versus None *€*2.852). When outpatient pharmacy costs were excluded from the overall costs, there were no differences in terms of the costs among the different types of treatments.

## 4. Discussion

Current retrospective analysis of a nationwide sample of Turkish patients not only provides solid data for economic evaluation in Turkey but also contributes to the general knowledge of how costs are distributed across health care services for AS. This study provides information regarding real-world clinical practices across patient subgroups and includes variations difficult to assess using data from trials, surveys, expert opinions, and other primary data sources.

To our knowledge, this is the first study to investigate total medical health care costs associated with AS in Turkey using nationwide data. The only prior studies to analyze AS costs in Turkey were based on expert opinion, in which estimated direct costs were approximately *€*3.566 [23]. Other studies examined the epidemiology of AS [8, 24–26].

Recently, health care costs of AS has been calculated at *€*4.634 in Spain [12], *€*4.578 in Canada [15], and €3.676 in Germany [13]. Economic outcomes are not only influenced by patient and disease characteristics but also by international differences in medical practice as well as the financing and organization of health care and social security systems [27]. Therefore, estimates from other countries may not be easily transferable. 

In Turkey, payment by health insurance fund is based both on a retrospective fee-for-service (FFS) and a bundled payment system depending on the disease category and services related to the particular disease. Payment procedures are outlined by health budget laws (SUT). For example, private hospitals are generally paid according to the bundled payment system. University hospitals, however, are based on the FFS system. Laboratory services can be paid separately from the bundled payment system based on certain conditions. Access to biological drugs is determined by protocols of the Ministry of Health, and payment is determined by the health budget law of the SSI [28]. These protocols and health budget laws describe under what conditions, how much, and who should be prescribed these medications. 

This study's estimates were similar to other countries' estimated AS costs. Combining the AS incident and prevalent cases, annual health care costs of AS were *€*3.833 in Turkey. Approximately 88% of the overall costs were associated with pharmaceutical costs. Inpatient costs were approximately 4%, and outpatient costs comprised 8% of the overall costs. The average annual copay amount was *€*22. These ratios are not surprising in light of the fact that health service fees such as hospital and physician costs are lower in Turkey relative to European countries. Prior medication use significantly affected the cost estimation.

A number of studies found that costs were driven by disease severity [2, 11, 12]. Since clinical functions were missing from the data, comorbidity index scores and prior comorbid conditions were used as a proxy for severity. If prior medications are not controlled in the model, costs increased for patients with high comorbidities. Annual costs for AS patients with Elixhauser scores of at least 2 were approximately *€*4.218, whereas costs for patients with a score of lower than 2 costs were only *€*2.254. However, after controlling for medication use, comorbidity burden did not affect overall health care costs. 

In the past, fewer and less expensive treatment options were available for AS patients. The recent introduction of biologic medications was a substantial development for AS treatment. However, these new efficacious treatment options are available at a high price. A British study estimated that the average annual cost for a newly approved biologic medications prescribed for AS treatment is *€*15.622 [29]. The increased annual cost effect of these treatments in nationwide sample in Turkey was determined by this study. The total annual cost of prevalent AS patients prescribed biologic medications only was *€*8.359. 

It is important to note that this study has limitations [30]. First, the analysis was based on claims data, which are collected for payment rather than research purposes. For example, the presence of a diagnosis code on a medical claim is not necessarily proof of the presence of disease because diagnoses may be incorrectly coded or included as rule-out criteria rather than actual disease. For example, many patients with nonradiographic axial SpA may have been recorded with the same ICD-10 codes as AS. In order to mitigate some of the problems associated with ICD-10 codes, detailed quality checks were applied, requiring two claims with ICD-10-CM diagnosis codes, per patient, to verify the AS diagnosis prior to the analysis. Diagnosis codes used to define comorbidities have over 90% specificity [31, 32].

Second, it is not uncommon in many parts of the world for mild AS patients who respond well to NSAIDs to buy these medications out-of-pocket while seeing their physicians in private practice and hospitals' settings only every few years. To the extent that these patients exist in our dataset, our cost estimates are underestimated since private practice claims would be missing. However, lower copayment amounts in Turkey mitigate this bias. 

Third, occurrence of a drug prescription fill does not guarantee actual consumption of the drug by the patient. Therefore, study results are biased to the extent that compliance rates are unevenly distributed among treatment options. Although a multivariate regression model was used to control for risk factors, the data did not contain any measure of disease activity, health status, or patient lifestyle. Comorbidity index scores, individual comorbidities, and baseline treatments were used to proxy for disease severity. However, they may not be a true representative of disease severity. Further studies can link the outcomes from claims data to hospital charts, where disease activity scores may be derived.

## 5. Conclusion

Nationally representative real-world data from Turkey was used to estimate health care costs associated with AS. To ensure that economic evaluations are relevant, country-specific observational studies are necessary. These study's findings are consistent with existing research and suggest that AS health care costs in Turkey resemble those in Europe. Costs are driven by treatment choices. The current data provide a baseline to evaluate the economic effect of such treatments and assist future AS-related health care policy and analysis. 

## Figures and Tables

**Table 1:** Demographic and clinical characteristics of patients with incident and prevalent ankylosing spondylitis.

	Incident		Prevalent		
	*N* = 603	Percentage (STD)	*N* = 2.383	Percentage (STD)	*P* value
Age (continuous)	38.74	11.55	40.52	11.35	0.0007
Age 18–39	352	58.37%	1221	51.24%	0.0017
Age 40–64	237	39.30%	1100	46.16%	0.0025
Age 65+	14	2.32%	62	2.60%	NS
Female	267	44.28%	905	37.98%	0.0046
Region					
Eastern Anatolia region	10	1.66%	44	1.85%	NS
Southeastern Anatolia region	14	2.32%	32	1.34%	0.0812
Marmara region	297	49.25%	1094	45.91%	NS
Aegean region	77	12.77%	400	16.79%	0.0162
Mediterranean region	41	6.80%	182	7.64%	NS
Black Sea region	34	5.64%	110	4.62%	NS
Central Anatolia region	130	21.56%	521	21.86%	NS
Comorbidities					
Elixhauser index score	2.24	3.89	6.04	5.01	<0.0001
Cardiovascular	67	11.11%	390	16.37%	0.0014
Diabetics	36	5.97%	192	8.06%	0.0847
Respiratory	144	23.88%	896	37.60%	<0.0001
Allergy	46	7.63%	148	6.21%	NS
Medications					
TNF	45	7.46%	828	34.75%	<0.0001
NSAIDs	603	71.64%	2383	77.01%	0.0055
DMARDs	129	21.39%	555	23.29%	NS

STD: standard deviation; NS: not significant; TNF: tumor necrosis factor; NSAIDs: nonsteroidal anti-inflammatory drugs; DMARDs: disease-modifying antirheumatic drugs.

**Table 2:** Annual cost of incident and prevalent ankylosing spondylitis patients in Turkey.

	Incident		Prevalent	
	Mean	Overall %	Mean	Overall %
Inpatient cost	€161.51	7.17	€154.7	3.65
Outpatient cost	€331.41	14.71	€297.43	7.03
Pharmacy cost	€1.737.62	77.1	€3.760.39	88.83
Copays	€23.09	1.02	€20.63	0.49
Overall	€2.253.63	100	€4.233.15	100

**Table 3:** Marginal effects calculated after generalized linear model estimation.

	Prevalent cases		Incident cases	
	€	*P* value	€	*P* value
Age 18–39	−320	0.227	−1040	0.045
Age 40–64	−155	0.555	−673	0.144
Age 65+	Reference		Reference	
Female	−70	0.456	−177	0.332
Region				
Eastern Anatolia region	226	0.622	402	0.707
Southeastern Anatolia region	Reference		Reference	
Marmara region	−203	0.549	−282	0.740
Aegean region	−204	0.481	−633	0438
Mediterranean region	−350	0.312	−513	0.534
Black Sea region	−150	0.698	−178	0.839
Central Anatolia region	48	0.890	−13	0.987
Elixhauser index ≥2	129	0.580	− 96.57	0.669
Individual comorbidities				
Diabetics	184	0.238	−46	0.906
Respiratory	288	0.002	168	0.428
Allergy	−187	0.336	−234	0.442
Cardiovascular	131	0.234	320	0.243
Medications				
Biologics	6258	0	5518	0.000
NSAIDs	−53	0.588	−263	0.239
DMARDs	−56	0.599	101	0.653

NSAIDs: nonsteroidal anti-inflammatory drugs; DMARDs: disease-modifying antirheumatic drugs.
